# The Significance of Utilizing A Corticotomy on Periodontal and Orthodontic Outcomes: A Systematic Review and Meta-Analysis

**DOI:** 10.3390/biology10080803

**Published:** 2021-08-19

**Authors:** Jonathan Gao, Trung Nguyen, Snehlata Oberoi, Heesoo Oh, Sunil Kapila, Richard T. Kao, Guo-Hao Lin

**Affiliations:** 1 Department of Orofacial Sciences, School of Dentistry, University of California San Francisco, San Francisco, CA 94131, USA; jonathan.gao@ucsf.edu (J.G.); trung.nguyen@ucsf.edu (T.N.); sneha.oberoi@ucsf.edu (S.O.); sunil.kapila@ucsf.edu (S.K.); rkao@ucsf.edu (R.T.K.); 2 Department of Orthodontics, Arthur A. Dugoni School of Dentistry, University of the Pacific, San Francisco, CA 94103, USA; hoh@pacific.edu; 3 Private Practice, San Jose, CA 95125, USA

**Keywords:** orthodontics, acceleration, periodontics, tooth movement technique, bone remodeling

## Abstract

**Simple Summary:**

The collaboration between periodontics and orthodontics has the potential to allow safer orthodontic tooth movement and improve vulnerable oral conditions especially for patients with very thin bone and soft tissue. By providing this interdisciplinary surgical approach where thin bone and soft tissue are surgically augmented to convert a fragile-thin to a robust-thick tissue phenotype, this permits orthodontic treatment in these previously thin tissue cases to proceed without iatrogenically induced adverse effects. This is an infrequently performed procedure with few clinical recommendations. This review paper provides the rationale and the currently available evidence on the benefits of this novel surgical approach.

**Abstract:**

Purpose: This systematic review compares the clinical and radiographic outcomes for patients who received only a corticotomy or periodontal accelerated osteogenic orthodontics (PAOO) with those who received a conventional orthodontic treatment. Methods: An electronic search of four databases and a hand search of peer-reviewed journals for relevant articles published in English between January 1980 and June 2021 were performed. Human clinical trials of ≥10 patients treated with a corticotomy or PAOO with radiographic and/or clinical outcomes were included. Meta-analyses were performed to analyze the weighted mean difference (WMD) and confidence interval (CI) for the recorded variables. Results: Twelve articles were included in the quantitative analysis. The meta-analysis revealed a localized corticotomy distal to the canine can significantly increase canine distalization (WMD = 1.15 mm, 95% CI = 0.18–2.12 mm, *p* = 0.02) compared to a conventional orthodontic treatment. In addition, PAOO also showed a significant gain of buccal bone thickness (WMD = 0.43 mm, 95% CI = 0.09–0.78 mm, *p* = 0.01) and an improvement of bone density (WMD = 32.86, 95% CI = 11.83–53.89, *p* = 0.002) compared to the corticotomy group. Conclusion: Based on the findings of the meta-analyses, the localized use of a corticotomy can significantly increase the amount of canine distalization during orthodontic treatment. Additionally, the use of a corticotomy as a part of a PAOO procedure significantly increases the rate of orthodontic tooth movement and it is accompanied by an increased buccal bone thickness and bone density compared to patients undergoing a conventional orthodontic treatment.

## 1. Introduction

A corticotomy is an in-office surgical procedure where decortication occurs in the dentoalveolar cortical bone with a degree of penetration into the medullary spaces [1]. Corticotomy procedures can be utilized in localized areas such as distal to the canine and anterior sextant or in a generalized manner. Corticotomy-assisted orthodontic treatment (CAOT) is a combination of bone activation through selective decortication and orthodontic forces [2,3] that utilizes a corticotomy in a generalized manner. Numerous clinical trials have shown that CAOT can result in a decreased treatment time, an enhanced resolution of crowding, the acceleration of canine distalization, a facilitated eruption of impacted teeth, improved molar intrusion, open bite correction, anchorage control and increased post-orthodontic stability [2].

The underlying concept of CAOT stems from initiating a regional acceleratory phenomenon, which was first described by Harold Frost [4]. A regional acceleratory phenomenon refers to a localized osteoporosis state, as part of the healing process, that can expedite hard and soft tissue healing two- to ten-fold [2]. Following a fracture, arthrodesis, an osteotomy or a bone grafting procedure, a regional acceleratory phenomenon often occurs by recruiting and activating the precursor cells necessary for wound healing concentrated at the injury site. While CAOT can accelerate tooth movement, it does not resolve the concern of the potential violation of the bony envelope when teeth are orthodontically moved. The concept of a corticotomy evolved to include alveolar ridge augmentation. This was first introduced as accelerated osteogenic orthodontics [1]. Accelerated osteogenic orthodontics consisted of buccal and lingual full-thickness flaps, a selective partial decortication of the cortical plates, concomitant bone grafting and augmentation and the primary flap closure [1]. Orthodontic reactivations and adjustments were performed every two weeks following surgery [1]. In the early years of accelerated osteogenic orthodontics, the bone augmentation was performed using either demineralized freeze-dried bone allograft (DFDBA) and bovine bone or bioactive glass [1]. In recent years, the use of 100% allograft or a mixture of DFDBA and bovine bone became preferred [3]. Most recently, accelerated osteogenic orthodontics has become known as periodontal accelerated osteogenic orthodontics (PAOO). The preferred grafting materials for PAOO include deproteinized bovine bone, autogenous bone, an allogeneic graft or a combination thereof [2]. The addition of bone grafting material increases the bone thickness and density [5]. This added osseous augmentation step is indicated in orthodontic situations where there is a concern that the orthodontic movement may move the tooth out of the bony housing resulting in bony defects such as fenestration and/or dehiscence as well as gingival recession. These negative outcomes may take years before they become clinically observable. PAOO has been proposed to alter the periodontal phenotype to prevent these negative outcomes [6].

A previous meta-analysis of a corticotomy performed during orthodontic treatment showed its effectiveness in accelerating maxillary canine distalization, treating anterior open bites with a skeletal anchorage and reducing an orthodontic relapse [7]. Additionally, the meta-analysis demonstrated that performing a corticotomy does not damage dental and periodontal structures. With the addition of bone grafting materials to the corticotomy procedure, studies [3,8] reported the potential benefits of preserving the periodontal structures surrounding teeth treated with PAOO. However, a comprehensive systematic review analyzing the induction of a regional acceleratory phenomenon on orthodontic outcomes is still lacking. Therefore, the aim of this review was to compare the clinical and radiographic outcomes for patients who received an orthodontic treatment with a localized corticotomy, anterior CAOT or PAOO to the ones who received a conventional orthodontic treatment. Specifically, using published prospective and retrospective studies meeting the inclusion criteria, this project assessed the rate of tooth movement and changes to the periodontal parameters of a localized corticotomy, anterior CAOT or PAOO procedure relative to a conventional orthodontic treatment.

## 2. Materials and Methods

### 2.1. Population, Intervention, Comparison, Outcome (PICO) Question 

The focused PICO [9] question was “Does the use of corticotomy procedures provide better clinical and radiographic outcomes than conventional treatments in patients who receive orthodontic treatment?”. The population selected was subjects receiving orthodontic treatment for correcting a malocclusion. The intervention investigated was the use of a corticotomy procedure (a localized corticotomy, anterior CAOT or PAOO) during orthodontic treatment. The primary outcomes to be compared were the rate of tooth movement and changes of periodontal parameters.

### 2.2. Selection Criteria

Human case-control studies or randomized controlled trials (RCTs) published in English between January 1980 and June 2021 were examined. The inclusion criteria were: (a) including ≥10 subjects receiving orthodontic treatment and having a corticotomy with or without bone grafting material and (b) reporting outcomes of one of the clinical (the amount of canine distalization, loss of molar anchorage, keratinized tissue gain, bone thickness gain, pocket depth reduction) and/or radiographic parameters (root length reduction, bone density change) after the treatment. Other types of articles such as editorials, letters or commentaries, animal/in vitro studies, literature reviews and case reports/series with <10 patients were excluded.

### 2.3. Screening Process

Two examiners (J.G. and G.L.) performed the literature search independently utilizing four databases (Ovid MEDLINE, EMBASE, Web of Science and Cochrane Central). The search terms used in MEDLINE/PubMed were: ((“orthodontics” [MeSH] OR “orthodontic” [All fields]) AND (“corticotomy” [All fields] OR “grafting” [All fields] OR “accelerated” [All fields] OR “augmented” [All fields] OR “osteogenesis” [MeSH] OR “osteogenic” [All fields])).

For the other databases, the key terms used for the search included orthodontic, corticotomy, grafting, accelerated, augmented and osteogenic. In addition, a hand search in the references of related systematic reviews was performed to identify any publications that were not electronically accessible. The eligibility of the pre-identified articles was confirmed by the two reviewers (J.G. and G.L.) after a full-text review. The level of agreement between the two reviewers was analyzed with kappa statistics.

### 2.4. Data Extraction

The data from the included articles were extracted by two independent reviewers (J.G. and T.N.). If there were any disagreements between the reviewers, the disagreements were reconciled after a discussion with the third reviewer (G.L.). For each selected study, demographic data such as the study design, sample size, numbers of participants, follow-up period, treatment outcome measurements and study conclusion were extracted and recorded.

### 2.5. Data Analyses

The primary outcomes were the amount of canine distalization and loss of molar anchorage and the secondary outcomes were the changes in the recorded clinical and radiographic parameters. The pooled weighted mean difference (WMD) and the 95% confidence interval (CI) of the recorded variables were analyzed using a computer program (RevMan Version 5.0, The Nordic Cochrane Centre, The Cochrane Collaboration, Copenhagen, Denmark, 2008). Heterogeneity was evaluated with a chi-squared test and an I^2^ test, which ranged between 0% and 100% with the lower values representing less heterogeneity. Fixed effects meta-analyses were applied if the pooled studies presented with a low heterogeneity; however, if a high heterogeneity was identified, random effects meta-analyses were applied to minimize bias caused by methodological differences. Forest plots were produced to represent the WMD and 95% CI of the primary and secondary outcomes. This study was registered at the PROSPERO database (https://www.crd.york.ac.uk/PROSPERO/display_record.php?RecordID=199826, accessed on 8 August 2021) under the registration code CRD42020199826. The reporting of the meta-analyses adhered to the PRISMA (Preferred Reporting Items for Systematic Review and Meta-Analyses) statement [10].

### 2.6. Risk of Bias Assessment

The Randomized Clinical Trial Checklist of the Cochrane Center [11] criteria were used to assess the methodological aspects of the included RCTs. The degree of bias was categorized as low, high or uncertain risk [11]. Additionally, the risk of bias of the included case-control studies was evaluated using the Newcastle–Ottawa Scale [12]. Each case-control study was assessed and could be awarded a maximum of nine stars. Two reviewers (J.G. and T.N.) rated all the included studies independently.

## 3. Results

**Figure 1:**
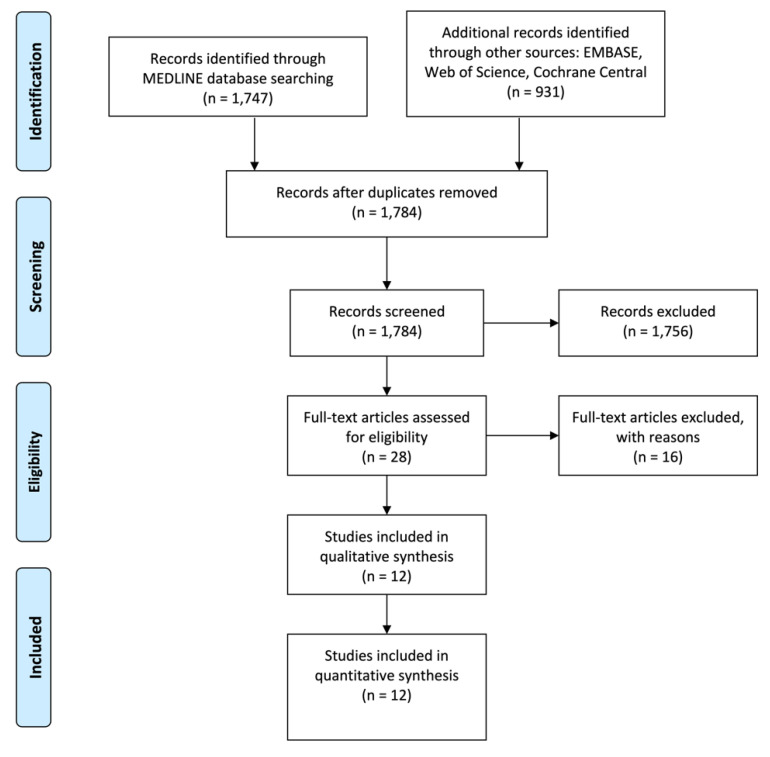
Flowchart illustrating the publication selection process.

As depicted in Figure 1, electronic and hand searches yielded 1784 articles, of which 28 articles were selected for a full-text evaluation after screening their titles and abstracts. Sixteen articles [13,14,15,16,17,18,19,20,21,22,23,24,25,26,27,28] were further excluded (Table 1); the reasons for exclusion were as follows: no data on comparing groups with and without corticotomy procedures, no control group or inadequate data to be pooled in meta-analyses. Twelve articles [8,29,30,31,32,33,34,35,36,37,38,39] were included in this systematic review and meta-analysis. Of the twelve articles, seven [29,30,31,32,33,35,36] compared a localized corticotomy (distal to the canine) to a conventional orthodontic treatment; three articles [8,38] compared PAOO to a conventional orthodontic treatment and the other two articles [34,37] compared anterior CAOT to PAOO. The main features and conclusions of the included studies are summarized in Table 2.

The kappa value for the inter-reviewer agreement for potentially relevant articles was 0.88 (titles and abstracts) and 0.92 (full-text articles), indicating an “almost perfect” agreement between the two reviewers [40].

### 3.1. Features of the Included Studies

#### 3.1.1. Study Design and Participant Features

Eight RCTs [30,31,32,33,34,35,36,37] and four case-control studies [8,29,38,39] were included in this systematic review. The age of the participants ranged from 12 to 61 years of age [38]. Two studies consisted of an Angle Class I malocclusion [34,37], five studies with a Class II [29,31,32,33,36] two studies with a Class III [8,39] and three studies did not report the classification of the malocclusion [30,35,38].

#### 3.1.2. Outcome Measurements

All seven studies [29,30,31,32,33,35,36] that compared a localized corticotomy to a conventional orthodontic treatment reported an outcome on canine distalization. Six [29,30,31,33,35,36] out of the seven studies also reported the amount of molar anchorage loss. For the studies that compared PAOO to a conventional orthodontic treatment, two studies reported outcomes on the amount of keratinized tissue gain [1,39] and bone thickness gain [8,39]. For the studies that compared PAOO to anterior CAOT, both studies [34,37] reported outcomes on the pocket depth reduction, root length reduction and bone density change.

#### 3.1.3. Anatomic Location of the Study Sites

In all studies [29,30,31,32,33,35,36] that compared a localized corticotomy to a conventional orthodontic treatment, the distal aspect of the canines was the study site. In three studies [8,38,39] that compared PAOO to a conventional orthodontic treatment, two studies [8,38] focused on mandibular anterior teeth while the third [39] looked at maxillary anterior teeth. In two studies [34,37] that compared anterior CAOT to PAOO, the mandibular anterior teeth were the site of interest.

#### 3.1.4. Active Treatment Time for Patients Receiving Anterior CAOT or PAOO

For the studies that compared anterior CAOT to a conventional orthodontic treatment, the active treatment time ranged from 1 [29] to 4–5 months [36] after the corticotomy surgery. For the studies that compared PAOO to a conventional orthodontic treatment, the active treatment time ranged from 7.1 [38] to 8.7 months [8]. On the contrary, the treatment time of the conventional orthodontic group ranged from 10.9 [38] to 22.1 [8] months with one other study [39] that did not report the active treatment time. Regarding the studies that compared PAOO to CAOT, the PAOO treatment time ranged from 14.4 [34] to 16.8 weeks [34]. In comparison, the treatment time of the anterior CAOT group was 15 [37] to 17 weeks [34]. The PAOO group demonstrated consistently a reduced active treatment time compared to the conventional orthodontic treatment but not the anterior CAOT group.

#### 3.1.5. Bone Grafting Materials

In addition to the corticotomy procedure, bone grafting material was used during surgical treatment in various studies performing PAOO. For the studies that compared PAOO to conventional orthodontics, various bone grafting materials were used including DFDBA [38], bovine bone xenografts [8,38] and a tricalcium phosphate bone substitute [39]. For the studies that compared PAOO to anterior CAOT, bioactive glass was used in both studies for the PAOO procedure [34,37]. Bahamman [34] also introduced another study arm and compared the results from a bovine bone xenograft to a bioactive glass augmentation with the conventional orthodontic treatment as the control. Other studies that compared a localized corticotomy to a conventional orthodontic treatment did not utilize bone grafting material [29,30,31,32,33,35,36].

#### 3.1.6. Type of Localized Corticotomy

For studies comparing a localized corticotomy to a conventional orthodontic treatment, micro-osteoperforation was used in five studies [30,31,33,35,36]. Among the five studies, one study used piezocision for a micro-osteoperforation [31] and three studies used mini-screws [30,33,35]. The fifth study [36] used a standardized needle gun to perform the micro-osteoperforation. The other two studies [29,32] performed a full-thickness flap to facilitate a localized corticotomy using a high-speed handpiece.

### 3.2. Meta-Analyses for the Outcomes of a Localized Corticotomy Compared to a Conventional Orthodontic Treatment

**Figure 2:**
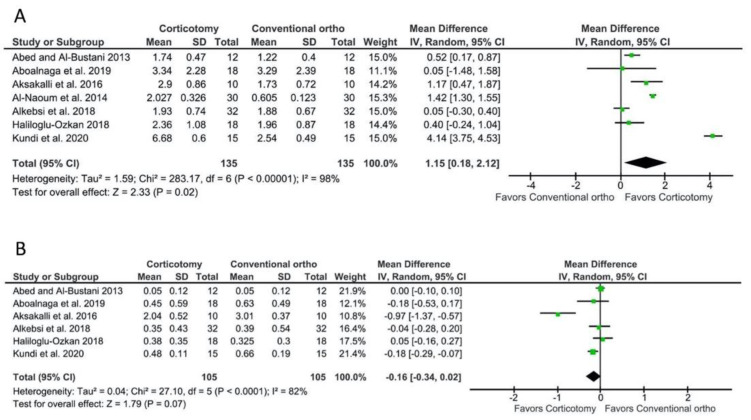
Meta-analyses for the outcomes of a localized corticotomy (distal to the canine) compared to a conventional orthodontic treatment. (**A**) For canine distalization, the WMD was 1.15 mm (95% CI = 0.18–2.12 mm, *p* = 0.02), favoring the corticotomy group. (**B**) For molar anchorage loss, the WMD was −0.16 mm (95% CI = −0.34–0.02 mm, *p* = 0.07), representing no statistically significant difference between the groups.

Seven studies [29,30,31,32,33,35,36] reported data on the amount of canine distalization between patients receiving a localized corticotomy and a conventional orthodontic treatment. The meta-analysis showed a WMD of 1.15 mm (95% CI = 0.18–2.12 mm, *p* = 0.02; Figure 2A), favoring the localized corticotomy group. However, the comparison presented a high heterogeneity among studies; the I^2^ test was 98% with a *p*-value < 0.0001 for the chi-squared test.

Six studies [29,30,31,33,35,36] reported data on the amount of molar anchorage loss. The meta-analysis showed a WMD of −0.16 mm (95% CI = −0.34–0.02 mm, *p* = 0.07; Figure 2B), representing no statistically significant difference between the groups. The comparison also presented a high heterogeneity among studies; the I^2^ test was 82% with a *p*-value < 0.0001 for the chi-squared test.

### 3.3. Meta-Analyses for the Outcomes of PAOO Compared to a Conventional Orthodontic Treatment

**Figure 3:**
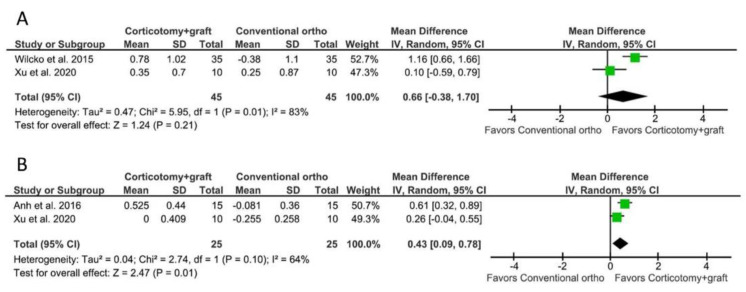
Meta-analyses for the outcomes of PAOO compared to a conventional orthodontic treatment. (**A**) For the gain of keratinized tissue width, the WMD was 0.66 mm (95% CI = −0.38–1.70 mm, *p* = 0.21), representing no statistically significant difference between the groups. (**B**) For the gain of bone thickness, the WMD was 0.43 mm (95% CI = 0.09–0.78 mm, *p* = 0.01), favoring the PAOO group.

Two studies [38,39] reported data on the amount of keratinized tissue gain between patients receiving PAOO and a conventional orthodontic treatment. The meta-analysis showed a WMD of 0.66 mm (95% CI = −0.38–1.70 mm, *p* = 0.21; Figure 3A), representing no statistically significant difference between the groups. The comparison presented a high heterogeneity among studies; the I^2^ test was 83% with a *p*-value for the chi-squared test of 0.01.

Two studies [8,39] reported data on the gain of bone thickness. The meta-analysis showed a WMD of 0.43 mm (95% CI = 0.09–0.78 mm, *p* = 0.01; Figure 3B), favoring the PAOO group. However, the comparison presented a high heterogeneity among studies; the I^2^ test was 64% with a *p*-value for the chi-squared test of 0.10.

### 3.4. Meta-Analyses for the Outcomes of Anterior CAOT Compared to PAOO

**Figure 4:**
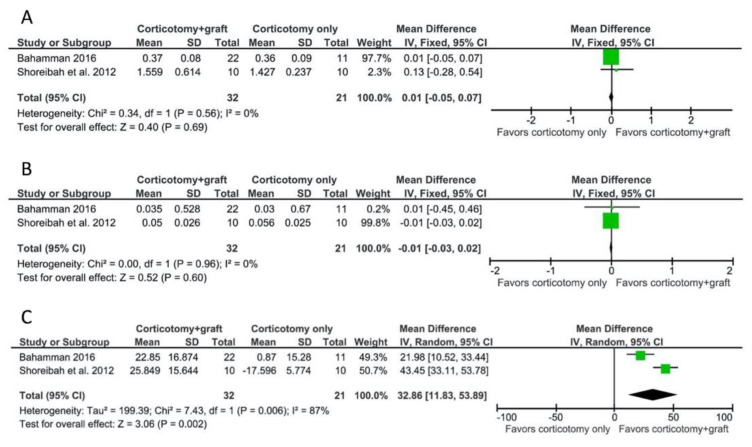
Meta-analyses for the outcomes of anterior CAOT compared to PAOO. (**A**) For pocket depth reduction, the WMD was 0.01 mm (95% CI = −0.05–0.07 mm, *p* = 0.69), representing no statistically significant difference between the groups. (**B**) For root length reduction, the WMD was −0.01 mm (95% CI = −0.03–0.02 mm, *p* = 0.60), representing no statistically significant difference between the groups. (**C**) For the change of bone density, the WMD was 32.86 (95% CI = 11.83–53.89, *p* = 0.002), favoring the PAOO group.

Two studies [34,37] reported data on pocket depth reduction and root length reduction between patients receiving PAOO and CAOT. The meta-analysis showed a WMD of 0.01 mm (95% CI = −0.05–0.07 mm, *p* = 0.69; Figure 4A) and −0.01 mm (95% CI = −0.03–0.02 mm, *p* = 0.60; Figure 4B) for pocket depth reduction and root length reduction, respectively, representing no statistically significant difference between the groups. The comparison presented a low heterogeneity among the studies for both analyses (I^2^ test = 0% with a *p*-value for the chi-squared test of 0.56 for pocket depth reduction; I^2^ test = 0% with a *p*-value for the chi-squared test of 0.96 for root length reduction).

For the change of bone density, the WMD of the pooled studies [34,37] was 32.86 (95% CI = 11.83–53.89, *p* = 0.002; Figure 4C), favoring the PAOO group. However, the comparison presented a high heterogeneity among the studies; the I^2^ test was 87% with a *p*-value for the chi-squared test of 0.006.

### 3.5. Risk of Bias Assessment

The risk of bias evaluation for the RCTs is summarized in Table A1. Of the eight [30,31,32,33,34,35,36,37] included RCTs, one study [30] was ranked low for the risk of bias in every category. Three studies [31,32,36] were considered to have two categories with an uncertain risk of bias. One study [33] was identified with an uncertain risk of bias in one area and a high risk of bias in a second category. One study [34] had three categories with an uncertain risk of bias. Another study [35] had two categories with an uncertain risk of bias and a high risk of bias in another two categories. The last study [37] had five categories with an uncertain risk of bias.

The risk of bias assessment for the included four [8,29,38,39] case-control studies is summarized in Table A2. All four studies [8,29,38,39] were scored seven stars out of nine stars according to the Newcastle–Ottawa Scale [12] and, therefore, were determined to have a considerable risk of bias.

## 4. Discussion

A corticotomy is a surgical procedure that triggers bone remodeling and enables rapid tooth movement. It can be performed in a localized area, such as the distal aspect of the canine, or in multiple sites of the arch. During the corticotomy procedure, only the bony architecture and physiology are influenced and there is no change to the soft tissue parameters. Based on the results of our meta-analysis, canine distalization can be enhanced by performing a localized corticotomy while molar anchorage is not influenced by this intervention. Most of the included studies showed an increased amount of canine distalization with the exception of two studies [30,33]. The differential findings between the investigators may be explained by the heterogeneity of the inclusion and exclusion criteria of the patients. For example, Alkebsi et al. [33] excluded patients over the age of 16, which poses a potential patient selection bias. Another source of inconsistent results may be the surgical approach used for the corticotomy. Alkebsi et al. [33] suggested that the lack of a regional acceleratory phenomenon triggered by micro-osteoperforations using mini-screws might result in inadequate bone remodeling stimulation. However, another study [35] utilizing mini-screws to perform micro-osteoperforations considered it to be an effective method for increasing the rate of tooth movement. In addition, studies that utilized burs, piezocision or a standardized needle gun to perform micro-osteoperforations appeared to show more significant regional acceleratory phenomenon-associated orthodontic movements. Interestingly, it has also been argued that a triggered regional acceleratory phenomenon from one side can have a crossover effect on the contralateral side of the mouth, which may compromise the outcome of the studies that used a split-mouth design [33].

It is worth noting that canine distalization recorded in two included studies [33,35] was based on a cusp tip movement instead of an apex movement. The movement of cusp tips in these cases was more distal than the apices, suggesting canine distalization primarily from canine tipping [30]. Therefore, the movement of the apex is a topic of interest as the distalization may majorly result from tipping instead of a bodily movement. However, our study did not identify sufficient data to analyze the outcome of canine tipping. The impact of a localized corticotomy on canine tipping should be further investigated in the future.

Loss of molar anchorage was previously reported even with an absolute anchorage [41]. With the use of temporary anchorage devices, the mini-screws were found to be displaced in the direction of the orthodontic loading, resulting in the loss of an absolute anchorage [41]. However, based on our meta-analysis, we did not detect a significant anchorage loss when performing a corticotomy in a localized region. The studies pooled in our analysis found minimal anchorage loss from the localized corticotomy compared to the control group. Therefore, performing a localized corticotomy distal to the canine should have no difference in anchorage loss compared to a conventional orthodontic treatment.

PAOO follows the protocol of performing a corticotomy in several sites of the arch combined with bone grafting. The goal of PAOO is not only to decrease the treatment time after initiating a regional acceleratory phenomenon but also to improve the soft tissue and hard tissue outcomes. Most studies [8,38] comparing PAOO to conventional orthodontics concluded that there is a shortened treatment time, possibly attributable to the regional acceleratory phenomenon. A corticotomy allows for demineralization at surgical sites and the adjacent bone, causing an intensified bone response that enables localized soft and hard tissue remodeling. This phenomenon allows healing to occur 2–10 times more rapidly than physiological healing [34].

In addition to a shortened treatment time, the gain of keratinized tissue width and bone thickness has been described in the literature [3,39]. However, our meta-analysis did not find a statistically significant gain of keratinized tissue width. Although both studies pooled in the analysis [3,39] found a tendency toward keratinized tissue gain after PAOO, the small sample size may have contributed to the non-significant statistical difference. Whether the increased keratinized tissue width is secondary to the orthodontic extrusion and correction of crowding should also be investigated further in the future.

While there is little evidence of keratinized tissue gain with PAOO, a statistically significant gain in bone thickness was found in the PAOO group. In our analysis, the PAOO group resulted in a 0.43 mm thickness gain compared to the conventional orthodontic group. This difference may have resulted from the grafting material used in the PAOO procedure. Although this difference is small, in cases with a thin buccal bone, performing PAOO offers the benefit of converting the bone from thin to a more robust bone morphotype. This change can further facilitate a favorable outcome when the augmentation occurs with a corticotomy-enhanced tooth movement. Furthermore, the addition of bone grafting material may also reduce the risk of fenestration, dehiscence and gingival recession associated with the orthodontic treatment [39].

Two included studies [34,37] reported no significant difference in terms of pre-operative and post-operative pocket depth at the sites receiving either PAOO or CAOT procedures. Hence, a reduction in pocket depth after PAOO or CAOT procedures cannot be expected. In addition, there is no difference with regard to root resorption between the two groups. Therefore, both PAOO and CAOT procedures are considered to have a minimal effect on root resorption. This finding is consistent with an earlier report [42].

As performing CAOT in several sites of the alveolus may result in a regional acceleratory phenomenon throughout the decorticated region, a reduction in bone density is a concern due to the increased bone turnover at the sites of a corticotomy. Conversely, our meta-analysis showed the addition of bone grafting material following a PAOO protocol may improve the bone density compared to the CAOT group. This increase in bone density may be one of the biggest advantages for performing PAOO. Shoreibah et al. [37] showed a significant increase in bone density measured by the 25.849 increase in gray values in the PAOO group compared to the CAOT group where a 17.596 decrease in gray values was detected. Another study [34] found that bone density reduction was seen at the time of debonding. However, this temporary reduction of bone density would be regained during the remineralization process due to a significant increase in bone density in the grafted groups compared to the control group. Currently available evidence [34,37] suggests that the addition of a xenograft may provide the best outcome to increase bone density after a corticotomy. However, the data on using allogenic grafting material and its effect on bone density are lacking and should be further studied.

The review and meta-analyses present the following limitations. First, due to the stringent criteria used in our review, the number of included studies was relatively small (n = 12). Second, there is a high level of heterogeneity related to the study design and methodology. Third, the risk of bias of the included studies is moderate to high. Several RCTs [31,34,35,36,37] did not report their random sequence generation and the method for allocation concealment, making these studies a moderate to high risk of bias. Therefore, the results of the meta-analyses should be interpreted cautiously. Future well-standardized RCTs are required to minimize research and technical design discrepancies to better address inconsistencies following the PRISMA guidelines [10].

## 5. Conclusions

Our systematic review found that the use of a localized corticotomy distal to the canine increases the amount of canine distalization but does not increase molar anchorage loss compared to a conventional orthodontic treatment. Furthermore, when a PAOO procedure is implemented during orthodontic treatment, a greater gain of buccal bone thickness and a higher post-operative bone density can be achieved than treatment done without bone grafting procedures.

## Figures and Tables

**Table 1:** Summary of the excluded articles.

Reason for Exclusion	Author (Year)
No data on comparing groups with and without corticotomy procedures.	Heidbuchel et al., 1993 [20]
	Makki et al., 2015 [22]
	Medeiros et al., 2018 [23]
	Singh and Jayan 2019 [25]
	Wang et al., 2014 [27]
No control group	Alfawal et al., 2018 [14]
Inadequate data to be pooled in meta-analyses	Aboul-Ela et al., 2011 [13]
	Alikhani et al., 2013 [15]
	Bhattacharya et al., 2014 [16]
	Charavet et al., 2019 [17]
	Feizbakhsh et al., 2018 [18]
	Gibreal et al., 2019 [19]
	Lee et al., 2007 [21]
	Salman and Ali 2014 [24]
	Sun et al., 2019 [26]
	Wu et al., 2015 [28]

**Table 2:** Features of the included articles; MOP: micro-osteoperforation.

Localized Corticotomy (Limited to Distal of the Canine) Compared to Conventional Orthodontic Treatment											
Author/Year	Study Design	Duration; Gender; Age Range	Case Type	Treatment Groups (sample size)	Treatment Location	Outcomes					Conclusions
						Canine Distalization Mean (SD)	Loss of Anchorage Mean (SD)	Canine Tipping Mean (SD)	Active Treatment Time		
Abed and Al-Bustani 2013 [29]	Case-Control Study	1 month post-surgery; 8f, 4m; average 21.7	Class II	T: Flap Corticotomy (12) C: Conventional orthodontics (12)	Distal aspect of canine	T: 1.74 (0.47) C: 1.22 (0.40)	T: 0.05 (0.12) C: 0.05 (0.12)	Not reported	1 month		Flap corticotomy is effective in accelerating orthodontic tooth movement with no harmful effects on surrounding vital structures and/or pulp vitality.
Al-Naoum et al. 2014 [32]	RCT	3 months post-surgery; 15f, 15m; 20.04 (3.63)	Class II	T: Flap Corticotomy (30) C: Conventional orthodontics (30)	Distal aspect of canine	T: 2.027 (0.326) C: 0.605 (0.123)	Not reported	Not reported	3 months		Flap corticotomy increased orthodontic tooth movement. Velocities after corticotomies were 2 to 4 times faster in the test group compared to control.
Aksakalli et al. 2016 [31]	RCT	3.5 months post-surgery; 6f, 4m; 16.3 (2.4)	Class II	T: MOP with piezocision (10) C: Conventional orthodontics (10)	Distal aspect of canine	T: 2.90 (0.86) C: 1.73 (0.72)	T: 2.04 (0.52) C: 3.01 (0.37)	Not reported	3.5 months		MOP with piezocision-assisted distalization accelerates tooth movement, decreases the anchorage loss for posterior teeth, and does not induce any maxillary transversal change or adverse effects on periodontal heath.
Alkebsi et al. 2018 [33]	RCT	3 months post-surgery; 24f, 8m; 19.26 (2.48)	Class II	T: MOP with mini-screw (32) C: Conventional orthodontics (32)	Distal aspect of canine	T: 1.93 (0.74) C: 1.88 (0.67)	T: 0.35 (0.43) C: 0.39 (0.54)	T: 0.25 (0.23) C: 0.25 (0.26) Data in mm	3 months		MOP was not effective in accelerating tooth movement at any time point. There was no significant difference between test and control at any time points.
Haliloglu-Ozkan et al. 2018 [35]	RCT	2 months post-surgery; 13f, 19m; T: 15.27 (1.62), C: 16.13 (1.28)	Not reported	T: MOP corticotomy with mini-screw (18) C: Conventional orthodontics (18)	Distal aspect of canine	T: 2.36 (1.08) C: 1.96 (0.87)	T: 0.38 (0.35) C: 0.325 (0.30)	T: 7.57 (2.67) C: 4.545 (2.05) Data in degree	2 months		MOP did not facilitate accelerated canine distalization or loss of molar anchorage. Canine tipping was significant in the treatment group.
Aboalnaga et al. 2019 [30]	RCT	4 months post-surgery; 18f; 16–25	Not reported	T: MOP corticotomy with mini-screw (18) C: Conventional orthodontics (18)	Distal aspect of canine	T: 3.34 (2.28) C: 3.29 (2.39)	T: 0.45 (0.59) C: 0.63 (0.49)	The canine cusp tips moved a greater distance than the apices in both sides	4 months		MOP did not facilitate accelerate canine distalization. Also, it did not reduce molar anchorage compared to the control group.
Kundi et al. 2020 [36]	RCT	16 months post-surgery; 16f, 14m; 20–36	Class II	T: MOP corticotomy with standardized needle gun (15) C: Conventional orthodontics (15)	Distal aspect of canine	T: 6.68 (0.60) C: 2.54 (0.49)	T: 0.48 (0.11) C: 0.66 (0.19)	Not reported	4–5 months		MOP accelerated canine distalization. There was no significant difference in anchorage loss between treatment and control group.
**PAOO (Anterior Corticotomy + Graft) Compared to Conventional Orthodontic Treatment**											
Author/Year	Study Design	Duration; Gender; Age Range	Case Type		Treatment Groups (sample size)	Treatment Location	Outcomes				Conclusions
							Keratinized Tissue Gain Mean (SD)	Bone Thickness Gain Mean (SD)	Active Treatment Time		
Wilcko et al. 2015 [38]	Case-Control Study	T: 19.4 months post-treatment, C: 15.9 months post-treatment; 48f, 22m; 12.1–61.5	Not reported; no open bites included		T (PAOO): Corticotomy + DFDBA and bovine xenograft (35) C: Conventional orthodontics (35)	Mand ant	T: 0.78 (1.02)C: −0.38 (1.10)	Not reported	T: 7.1 (1.7) monthsC: 22.1 (6.8) months		PAOO helps in increasing keratinized tissue surrounding dentition compared to conventional orthodontic treatment.
Ahn et al. 2016 [8]	Case-Control Study	T: 8.7 months C: 10.9 months pre-orthognathic; 16f, 14m; T: 23.06 (6.16) C: 21.51 (3.34)	Class III		T (PAOO): Corticotomy + bovine xenograft (15) C: Conventional orthodontics (15)	Mand ant	Not reported	T: 0.525 (0.44) C: −0.081 (0.36)	T: 8.7 months C: 10.9 months		PAOO provided a favorable decompensation pattern for mandibular incisors and preserved the periodontal structures surrounding mandibular anteriors.
Xu et al. 2020 [39]	Case-Control Study	6 months post-treatment; 14f, 6m; 18–30	Class III		T (PAOO): Corticotomy + tricalcium phosphate bone substitute (10) C: Conventional orthodontics (10)	Max ant	T: 0.35 (0.77) C: 0.25 (0.87)	T: 0 (0.409) C: −0.255 (0.258)	Not reported		PAOO does not negatively affect periodontium and alveolar bone based on the findings of bone from the trial.
**PAOO (Anterior Corticotomy + Graft) Compared to CAOT (Anterior Corticotomy)**											
Author/Year	Study Design	Duration; Gender; Age Range	Case Type		Treatment Groups (sample size)	Treatment Location	Outcomes				Conclusions
							Pocket Depth Reduction Mean (SD)	Root Length Reduction Mean (SD)	Bone Density Change Gray Value (SD)	Active Treatment Time	
Shoreibah et al. 2012 [37]	RCT	6 months post-treatment; 16f, 4m; average 24.5	Class I		T (PAOO): Corticotomy + Bioactive glass (10) C (CAOT): Corticotomy only (10)	Mand ant	T: 1.559 (0.614) C: 1.427 (0.237)	T: 0.050 (0.026) C: 0.056 (0.025)	T: 25.849 (15.644) C: −17.596 (5.774)	T: 16.67 weeks C: 17 weeks	Performing corticotomy significantly reduced the total time of treatment. The incorporation of bone grafting material with corticotomy increased the alveolar bone density significantly.
Bahamman 2016 [34]	RCT	9 months post-treatment; 23f, 10m; 18–27	Class I		T1 (PAOO): Corticotomy + Bioactive glass (11) T2 (PAOO): Corticotomy + Bovine xenograft (11) C (CAOT): Corticotomy only (11)	Mand ant	T1: 0.37 (0.08) T2: 0.37 (0.08) T1/T2 combined: 0.37 (0.08) C: 0.36 (0.09)	T1: 0.03 (0.50) T2: 0.04 (0.58) T1/T2 combined: 0.035 (0.528) C: 0.03 (0.67)	T1: 13.71 (14.33) T2: 31.99 (14.45) T1/T2 combined: 22.85 (16.874) C: 0.87 (15.28)	T1: 14.4 weeks T2: 16.8 weeks C: 15 weeks	Combination of orthodontic treatment and corticotomy decreased the duration of active treatment. Use of PAOO approach provided superior benefits in terms of increased bone density.

## Data Availability

Data available on request due to restrictions.

## Appendix A

**Table A1:** Risk assessment of publication bias for the included RCTs.

Study	Random Sequence Generation	Allocation Concealment	Blinding of Participants and Personnel	Blinding of Outcome Assessment	Incomplete Outcome Data Addressed	Selective Reporting	Other Bias
Al-Naoum et al., 2014 [32]	Low	Low	Uncertain	Uncertain	Low	Low	Low
Aksakalli et al., 2016 [31]	Uncertain	Uncertain	Low	Low	Low	Low	Low
Alkebsi et al., 2018 [33]	Low	Low	High	Low	Low	Uncertain	Low
Haliloglu-Ozkan et al., 2018 [35]	Uncertain	Uncertain	Low	Low	High	High	Low
Aboalnaga et al., 2019 [30]	Low	Low	Low	Low	Low	Low	Low
Kundi et al., 2020 [36]	Uncertain	Uncertain	Low	Low	Low	Low	Low
Shoreibah et al., 2012 [37]	Uncertain	Uncertain	Uncertain	Uncertain	Low	Uncertain	Low
Bahamman 2016 [34]	Uncertain	Uncertain	Low	Low	Low	Uncertain	Low

**Table A2:** Newcastle–Ottawa Quality Assessment Scale of included case-control studies.

	Selection	Comparability	Exposure
Abed and Al-Bustani 2013 [29]	★★★	★	★★★
Wilcko et al., 2015 [38]	★★★	★	★★★
Ahn et al., 2016 [8]	★★★	★	★★★
Xu et al., 2020 [39]	★★★	★	★★★
